# Facile Fabrication of Hierarchically Porous Boronic Acid Group-Functionalized Monoliths With Optical Activity for Recognizing Glucose With Different Conformation

**DOI:** 10.3389/fchem.2022.939368

**Published:** 2022-06-08

**Authors:** Yan Wang, Luwei Zhang, Yu-I Hsu, Taka-Aki Asoh, Hiroshi Uyama

**Affiliations:** Department of Applied Chemistry, Graduate School of Engineering, Osaka University, Suita, Japan

**Keywords:** hierarchical pore, monolith, optical activity, helical polymer, α-glucose

## Abstract

At present, various materials based on helical polymers are nanoparticle or microsphere, which is not ease of use in practical application. Accordingly, facile preparation hierarchically porous monolith based on helical polymer needs to be developed. Herein, hierarchically porous boronic acid group-functionalized monoliths that exhibited optical activity were fabricated with a facile method based on crosslinking and polymerization-induced phase separation (CPIPS). Chiral substituted acetylene and achiral substituted acetylene with a boronic acid group were used as monomers. By regulating the composition of the pre-polymerization solution, the permeability and macropore size of the porous structure could be controlled. The hierarchically porous structure and large surface area were confirmed by scanning electron microscopy and nitrogen gas adsorption/desorption isotherms. In particular, the boronic acid functional group that can interact with a *cis*-diol group was successfully introduced on the skeleton surface of the monoliths. Further, the main chain of the copolymer that constituted the monoliths exhibited a high *cis* content and tacticity, and the monoliths showed good optical activity. Thus, the present study established a facile method to synthesize hierarchically porous boronic acid group-functionalized monoliths with optical activity *via* CPIPS, and the monoliths showed potential in recognition, separation, and adsorption of compound with chirality and *cis*-diol groups.

## Introduction

Phenylboronic acid (PBA) is a unique molecule that can specifically recognize compounds containing *cis*-diol groups, such as catechol, polysaccharides, and glycoproteins, *via* reversible covalent bonds with great research significance in the fields of environmental, food, and biological detection (James et al., 1996; Ma and Shi, 2014; Shen and Xia, 2014; Li et al., 2015; Yesilyurt et al., 2016; Lan and Guo, 2019). Under high-pH conditions, PBA can form covalent bonds with the *cis*-diol group-containing compounds, which dissociate under low pH yielding pristine PBA and the *cis*-diol group-containing compounds. Thus, PBA and its derivatives exhibit a broad range of application prospects in biosensors, drug delivery and release, enrichment, and imaging (Brooks and Sumerlin, 2016).

It is well known that macromolecules in organisms often have chirality, which is closely related to the normal life activities of the organisms (Dou et al., 2020). It has been suggested that developing chiral materials is of great significance to human life (Etayo and Vidal-Ferran, 2013). For instance, drugs with chirality are frequently applied in clinical treatments. Further, chiral substances are ubiquitous in nature, including in amino acids, polysaccharides, and proteins (Xing and Zhao, 2018). For example, almost all natural amino acids that constitute proteins are in the *L*-conformation, while almost all natural monosaccharides are in the *D*-conformation. Chiral compounds with the same conformation often tend to interact with each other. In most cases, a pair of enantiomers significantly differs in its pharmacological activity, metabolism, and toxicity *in vivo* (Tokunaga et al., 2018) thus, developing techniques to obtain a single enantiomer is critical for pharmaceutical applications. However, the pair is very similar in its physical and chemical properties, making it difficult to separate the individual enantiomers. Although it is evident that incorporating a PBA functional group and chirality is meaningful, there is limited research in this area.

Recently, a series of helical polymers with desirable optical activity was synthesized. Helical polymers have attracted much attention because of their unique chiral amplification derived from the high tacticity of the main chain (Shimomura et al., 2014; Yashima et al., 2016; Wang S. et al., 2021; Gu et al., 2021; Li and Deng, 2021). Thus, they have exhibited great potential in many fields, such as molecular recognition, asymmetric catalysis, and chiral separation. Currently, most of the materials based on helical polymers are nanoparticles (Zhao and Deng, 2016; Zhao et al., 2016; Núñez-Martínez et al., 2021) or microspheres, (Zhou et al., 2010; Liu et al., 2012; Zhang et al., 2012; Liang et al., 2016) which are inconvenient for practical applications. Therefore, developing monolithic materials based on helical polymers is necessary.

A hierarchically porous monolith as a novel material simultaneously contains two or more pore scales, including micropores (<2 nm), mesopores (2–50 nm), and macropores (>50 nm) (Ma et al., 2019). The presence of micropores and mesopores can provide a more active center and larger surface area, while macropores can accelerate mass transfer. These materials have several advantages, such as ease of use, a porous structure, faster mass transfer rate, and good stability. They have been widely used in applications such as separation, exploitation of energy resources, and catalysis (Hu et al., 2007; Fang et al., 2011; Hasegawa et al., 2011; Parlett et al., 2013; Nischang and Causon, 2016). Previously, we fabricated a hierarchically porous monolith with optical activity using crosslinking and polymerization-induced phase separation (CPIPS), which performed well in enantioselective crystallization.

In this study, we synthesized hierarchically porous boronic acid group-functionalized monoliths with optical activity using chiral and achiral substituted acetylenes as monomers to further develop the preparation method and introduce a functional group. Multiple characterizations were adopted to obtain the physical and chemical property of the monoliths. The synthesized monoliths have the potential in recognition, separation, and adsorption of substances with chirality and *cis*-diol groups.

## Materials and Methods

### Chemicals and Materials

Isobutyl chloroformate (≥98.0%), 4-carboxyphenylboronic acid (≥97.0%), 4-methylmorpholine (≥99.0%), (*R*)-(-)-2-phenylpropionic acid (≥98.0%), and (*S*)-(+)-2-phenylpropionic acid (≥98.0%) were purchased from Tokyo Chemical Industry Co., Ltd. (TCI, Tokyo, Japan). Bicyclo [2.2.1]hepta-2,5-diene-rhodium(I) chloride dimer (Rh (nbd)Cl_2_, ≥96%), propargylamine (≥98.0%), propargyl alcohol, and sodium tetraphenylboron were obtained from Sigma–Aldrich (St Louis, MO, United States). α-*D*- and α-*L*-Glucose were purchased from Thermo Fisher Scientific (Geel, Belgium). Anhydrous magnesium sulfate (MgSO_4_), benzene, sodium chloride (NaCl), sodium hydrogen carbonate (NaHCO_3_), 2 mol L^−1^ hydrochloric acid (HCl), p-toluenesulfonic acid monohydrate, super dehydrated tetrahydrofuran (THF), super dehydrated methanol (CH_3_OH), chloroform (CHCl_3_), and potassium bromide (KBr, infrared (IR) grade) were purchased from Wako Pure Chemical Industries, Ltd. (Wako, Osaka, Japan). Adipic acid (≥99.5%) and other solvents (tetrahydrofuran, 2-propanol, acetic ether, and ethanol) were acquired from Nacalai Tesque, Inc. (Kyoto, Japan).

### Synthesis of Chiral and Achiral Substituted Acetylene

Chiral substituted acetylene (*R-*monomer 1, *R-*M1; *S-*monomer 1, *S-*M1) and achiral substituted acetylene (monomer 2, M2) were synthesized according to a previously reported method (Deng et al., 2016; Lin et al., 2016). The chiral substituted acetylene was synthesized as follows: Briefly, *R-*M1 or *S-*M1 (10 mmol, 1.5 g) were dissolved in THF, followed by slowly dropping isobutyl chloroformate (10 mmol, 1.36 g) and 4-methylmorpholine (10 mmol, 1.01 g) in sequence. The mixture was reacted at 30°C while stirring for 40 min, after which propargylamine (10 mmol, 0.51 g) was added dropwise into the solution and stirred for 4 h at 30°C. Subsequently, the pale-yellow precipitate was removed by filtration to obtain the crude product, which was dissolved in acetic ether (100 ml). Then, 2 mol L^−1^ HCl (30 ml) and saturated NaHCO_3_ aqueous (30 ml) were used to wash the obtained solution three times, respectively. After drying the obtained product over anhydrous MgSO_4_ for 12 h, it was vacuum distilled to remove any solvent. Finally, the crude product was dissolved in THF (4 ml), which was dropped into a large amount of hexane for recrystallization to acquire the pure chiral substituted acetylene (*R-*M1 or *S-*M1). ^1^H NMR of *R-*M1 or *S-*M1 (400 MHz, CDCl_3_, δ ppm): 5.4 (s, 1H, −CH_2_NHCO−), 4.0 (m, 2H, CH≡CCH_2_NH−), 3.5 (m, 1H, −COCHCH_3_Ph), 2.2 (t, 1H, CH≡CCH−), 1.5 (t, 3H, −CH_3_).

The synthesis process of the achiral substituted acetylene (M2) is similar to that of chiral substituted acetylene with some modifications. 4-carboxyphenylboronic acid (10 mmol, 1.7 g) was selected as the precursor for the achiral substituted acetylene, while the other steps remained the same. ^1^H NMR of M2 (400 MHz, CDCl_3_, δ ppm): 8.9 (t, 1H, −CH_2_NHCO−), 8.1 (s, 2H, B(OH)_2_−), 4.0 (m, 2H, ≡CHCH_2_NH−), 3.1 (t, 1H, CH≡CCH−).

### Preparation of the Monolithic Materials

First, adipic acid and propargyl alcohol were used as precursors to synthesize the crosslinker according to a previously reported method (Nomura et al., 2003). [(nbd)Rh^+^B^−^(C_6_H_5_)_4_] was used as the catalyst, which was prepared using Rh (nbd)Cl_2_ and sodium tetraphenylboron (Chen et al., 2011). Then, the crosslinker (5.6 mg) and monomers (M1: 32.8 mg, M2: 15.2 mg) were weighed in tube A, and the catalyst (0.9 mg) in tube B. The two tubes were flushed with nitrogen to ensure an inert atmosphere and placed into a glove box. Then, super dehydrated CH_3_OH (232 μl) was added to tube A to dissolve the monomers and crosslinker, and super dehydrated THF (36 μl) was added to tube B to dissolve the catalyst. The two solutions were sufficiently mixed with each other and placed in a water bath at 15°C for 8 h (container: 6 × 40 mm glass tube). The obtained monolith was sufficiently washed with 2-propanol, ethanol, and deionized water in sequence to replace the previous solvents and remove any residue, followed by vacuum drying for 8 h.

### Selectively Chiral Adsorption Test

First, the 0.5 mg ml^−1^ α-*D*- and α-*L*-glucose were prepared using phosphate buffer (PB) solution (pH = 8.6) as solvent, and the monoliths to be tested were cut into slices. Subsequently, 30 mg monolith were added into 2 ml 0.5 mg ml^−1^ α-*D*- or α-*L*-glucose to shake for 12 h at 10°C without light. After adsorption, the monoliths were washed by 1% triethylamine (TEA) aqueous solution for three times to remove unabsorbed α-glucose and salt in PB solution, followed by vacuum drying for 8 h to test water contact angle.

### Instruments and Methods

Attenuated total reflectance IR (ATR-IR) spectra were obtained using Nicolet iS5 spectrometer outfitted with an iD5 ATR attachment (Thermo Scientific, Yokohama, Japan). The pore morphology of the hierarchically porous monolithic material was observed using scanning electron microscopy (SEM, 15 kV, SU-3500 instrument, Hitachi, Japan). The nitrogen adsorption/desorption isotherms were obtained using a surface area and pore size analyzer (NOVA 4200e, Quantachrome Instruments, United States). Density functional theory (DFT) was used to calculate the pore size distribution and pore volume. A thermogravimetric analyzer (STA7200RV, Hitachi, Japan) was employed to record the thermogravimetric analysis (TGA) curves by heating from 40 to 800°C under nitrogen atmosphere with a scanning rate of 10°C min^−1^. The Raman spectra were collected by a Raman spectrometer (NRS-3100, JASCO Corporation, Japan) with a 532 nm laser as an excitation source under 100 mW power. Circular dichroism (CD) spectroscopy and ultraviolet–visible (UV−vis) absorption spectrometry were performed using a spectropolarimeter (J-820 AC, JASCO Corporation, Japan). To determine the surface chemistry of the hierarchically porous monolith, the X-ray photoelectron spectroscopy (XPS, JEOL JPS-9010MC) was employed to characterize them with monochromatized Al-Kα radiation (1,486.6 eV). The analyzer pass energies were fixed at 30 eV for wide XPS spectra and 50 eV for narrow XPS spectra. The binding energies were referred to as C−H (sp3) carbon for the C 1s peak set at 284.6 eV. The contact angle was determined using a Drop Master DM300 (Kyowa Interface Science, Japan) with 1.0 μl solution drops.

According to Darcy’s law, the permeability of the porous materials could be calculated using the equation B_0_ = FηL/(πr^2^ΔP), where B_0_ is the permeability (m^2^), F is the flow rate of the mobile phase (m^3^ s^−1^), η is the viscosity of the mobile phase (Pa s), L is the effective length (m), r is the inner diameter (m), and ΔP is the pressure drop across the monolith (Pa). To measure the permeability, the obtained cylindrically-shaped monolith was tightly fitted with a proper heat shrink tube and connected to a digital pressure gauge (Krone, KDM30, Japan) to obtain ΔP, while a digital quantitative tubing pump (As One, DSP-100SA, Japan) was used to control the flow rate of the mobile phase.

## Results and Discussion

### Preparation of the Hierarchically Porous Boronic Acid Group-Functionalized Monolith With Optical Activity

**SCHEME 1:**
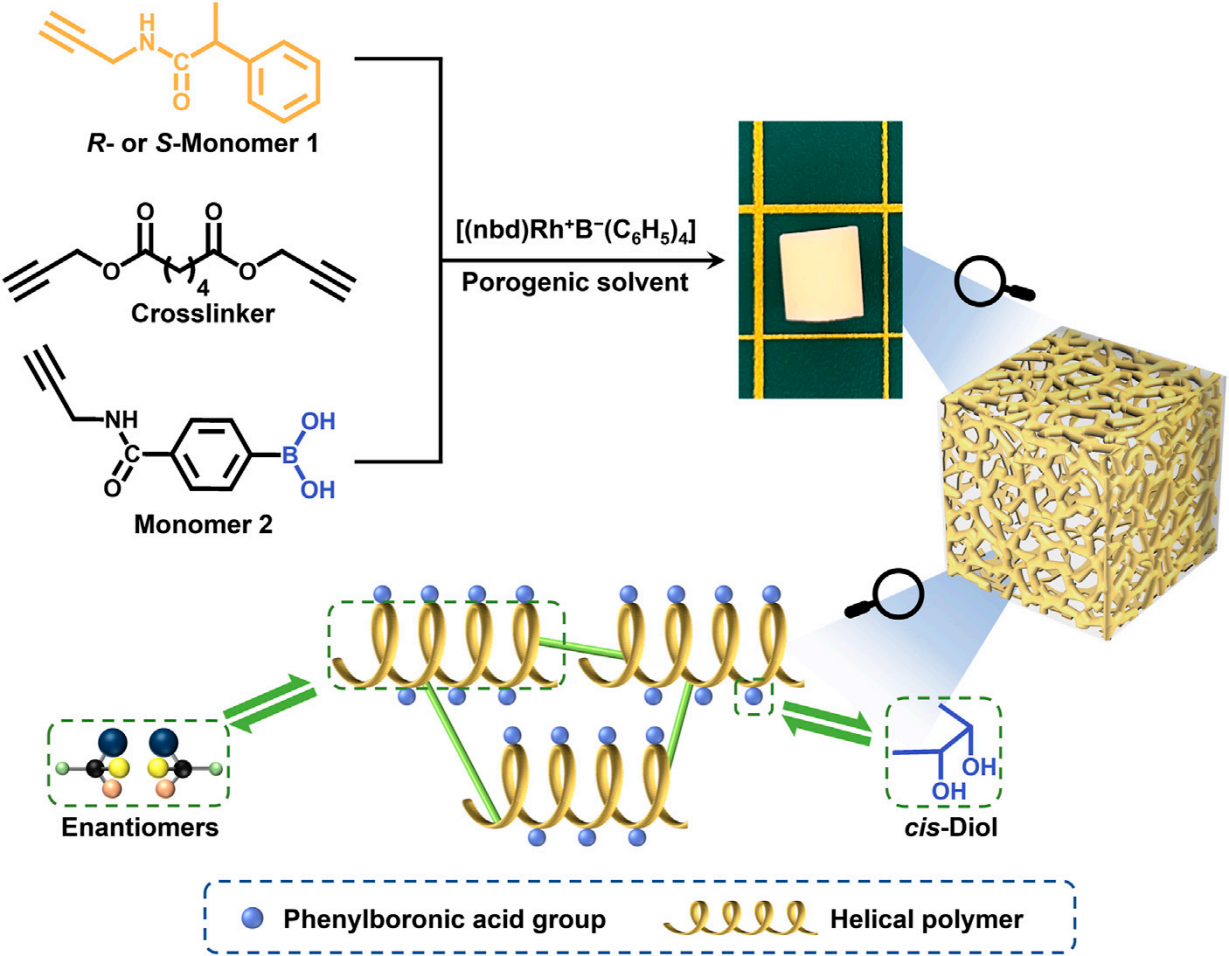
Strategy for preparing the hierarchically porous boronic acid group-functionalized monolith with optical activity *via* crosslinking and polymerization-induced phase separation.

In our previous study, we developed a flexible and highly efficient “one-step” method, the CPIPS method, to prepare chiral hierarchically porous monoliths (Wang Y. et al., 2021). To further develop this “one step” method and broaden its feasibility, we attempted to fabricate functionalized porous monoliths with optical activity using M1 with a chiral center, M2 with a boronic group, and a crosslinker as the raw materials. The helical polymer with chirality and the boronic acid functional group can interact with chiral and *cis*-diol group-containing compounds (Deng et al., 2016). The detailed preparation procedure is shown in Scheme 1.

**FIGURE 1:**
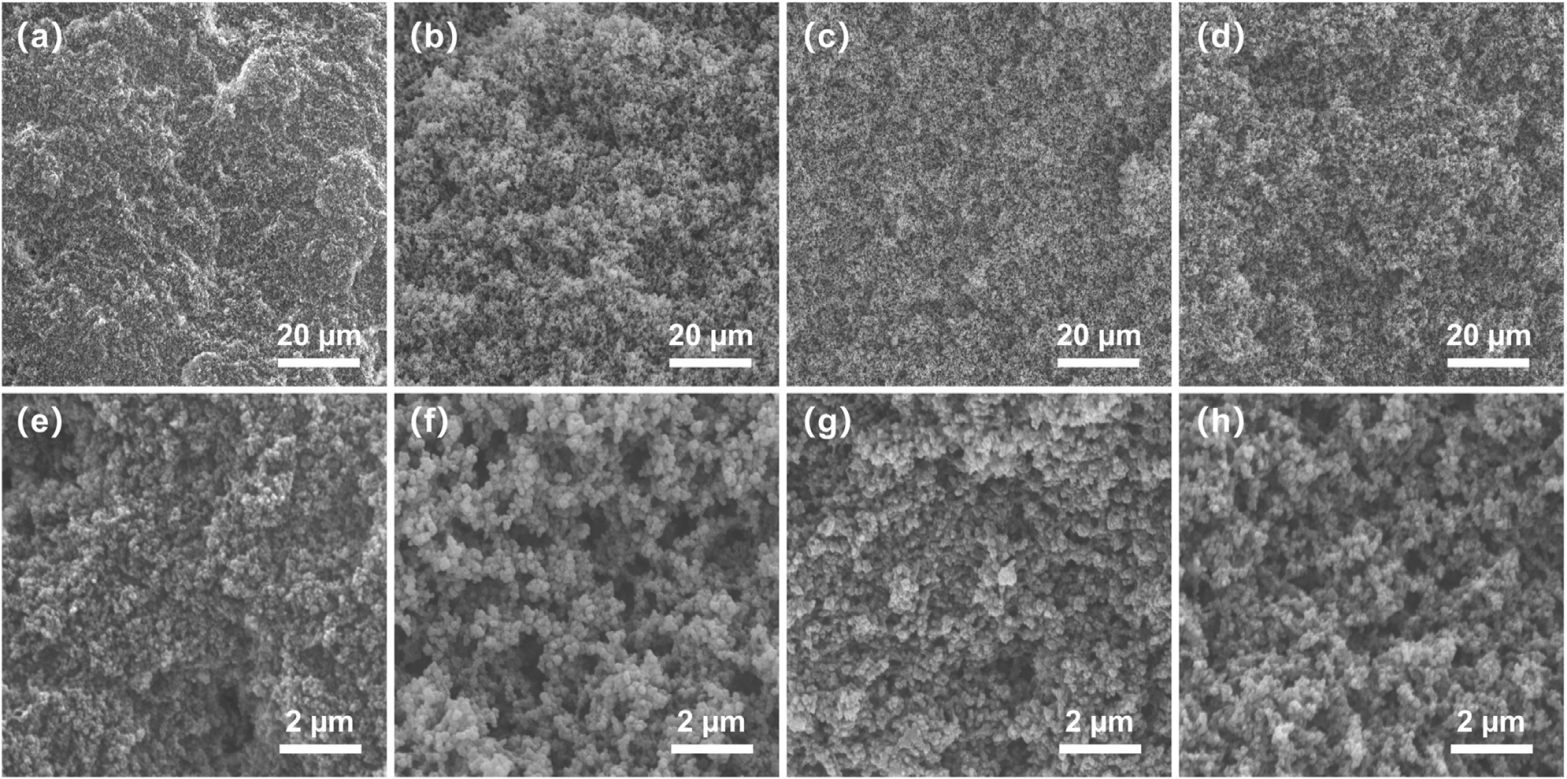
Scanning electron microscopy images of **(A,E)** monolith II, **(B,F)** monolith III, **(C,G)** monolith IV, and **(D,H)** monolith V. (a, b, c, and d ×2000 magnification, (d, e, f, and g ×10000 magnification.

For the preparation of porous monolith, the composition of the pre-polymerization solution and the proportion of porogenic solvent have a significant influence on its morphology; therefore, the preparation conditions were optimized as shown in Table 1. It has been suggested that the porogenic solvent plays a particularly important role in the formation of the monolith. Therefore, preliminary trials using different porogenic solvents, such as THF/polyethylene glycol (PEG) 200, THF/1-propanol, and THF/CH_3_OH, were conducted. When any proportion of THF/1-propanol or THF/PEG200 was used as porogenic system, the monolith cannot be formed, or there is no pore structure in monolith. Considering their porogenic ability and dissolving capacity, binary THF/CH_3_OH was eventually selected as the porogenic solvent for this study. The molar ratio of *R*-M1 and M2 was maintained at 3/1 to investigate the effect of the amount of THF/CH_3_OH on the monolith. As observed in Table 1, at 29.9% THF, the permeability of monolith II was 0.16 × 10^–14^ m^2^, which increased to 2.58 × 10^–14^ m^2^ at 13.4% THF for monolith III. Furthermore, SEM images of monolith II and III (Figures 1A,B,E,F) indicate that a lower content of CH_3_OH in the porogenic solvent yielded fewer large macropores. These results suggest that THF as the microporogenic solvent facilitates the formation of micropores and mesopores, while CH_3_OH is the macroporogenic component in the porogenic system.

**TABLE 1:** Composition of the polymerization mixtures and the permeability of the monoliths.

Monolith^a^	[*R*-M1]/[*S*-M1]/[M2] (mol/mol/mol)	THF^b^ (wt%)	CH_3_OH^b^ (wt%)	Total precursors Concentration (w/v, %)	Permeability (× 10^–14^ m^2^)
I	4/0/0	13.4	86.6	20	-^c^
II	3/0/1	29.9	70.1	20	0.16
III	3/0/1	13.4	86.6	20	2.58
IV	3/0/1	13.4	86.6	30	0.25
V	0/3/1	13.4	86.6	20	2.52
VI	0/0/4	13.4	86.6	20	-^d^

a The molar ratio of the total monomers/crosslinker in the pre-polymerization solution was maintained at 10/1, the molar ratio of total precursors/catalyst was kept at 157/1, and the reaction temperature was set to 15°C for 8 h.

b Weight percentage of the solvent in the porogenic mixture system.

c The monoliths easily fractured during measurement.

d The pressure drop was too high to measure permeability.

It was also observed that the concentration of the total precursors influenced the porous morphology and permeability of the monolith (Table 1). The permeability decreased from 2.58 × 10^–14^ m^2^ (monolith III) to 0.25 × 10^–14^ m^2^ (monolith IV) with an increase in the total precursor concentration. The SEM images of the two monoliths (Figures 1B,C,F,G) also confirmed the emergence of the larger macropores with the decrease of the total precursor concentration. As a result, a monolith with good permeability and more macropores could be fabricated in the presence of THF/CH_3_OH (v/v, 13.4/86.6) as the porogenic solvent and 20% total precursors concentration in the pre-polymerization solution. Hence, the permeability and macropore size of the prepared monolith could be adjusted by changing the composition of the pre-polymerization solution. Therefore, under the same conditions, monolith V was prepared using *S*-M1 as the monomer, yielding a good permeability of 2.52 × 10^–14^ m^2^, which was almost equal to that of monolith III. The same conditions were also employed to fabricate monolith I without M2 and monolith VI without M1. However, monolith I was fragile and fractured, while monolith VI was transparent without a porous structure; therefore, their permeability could not be measured. Because the raw materials in prepolymerization solution were changed, the optimized preparation conditions may not have been suitable for fabricating monoliths I and VI. To further investigate these differences, monoliths I, III, V, and VI were selected for subsequent characterization.

### Characterization of the Hierarchically Porous Boronic Acid Group-Functionalized Monolith With Optical Activity

Hierarchically porous materials combine the merits of micropores, mesopores, and macropores, thereby showing large potential in many fields (Sun et al., 2016; Wu et al., 2020). To confirm the hierarchically porous structures, the micro morphology of monoliths III and V was observed using SEM. Monoliths III and V showed a homogeneous and uniform porous structure, with macropores of approximately 1 μm, which can contribute to their permeability (Figures 1B,D,F,H); this is in agreement with the permeability results discussed above.

**FIGURE 2:**
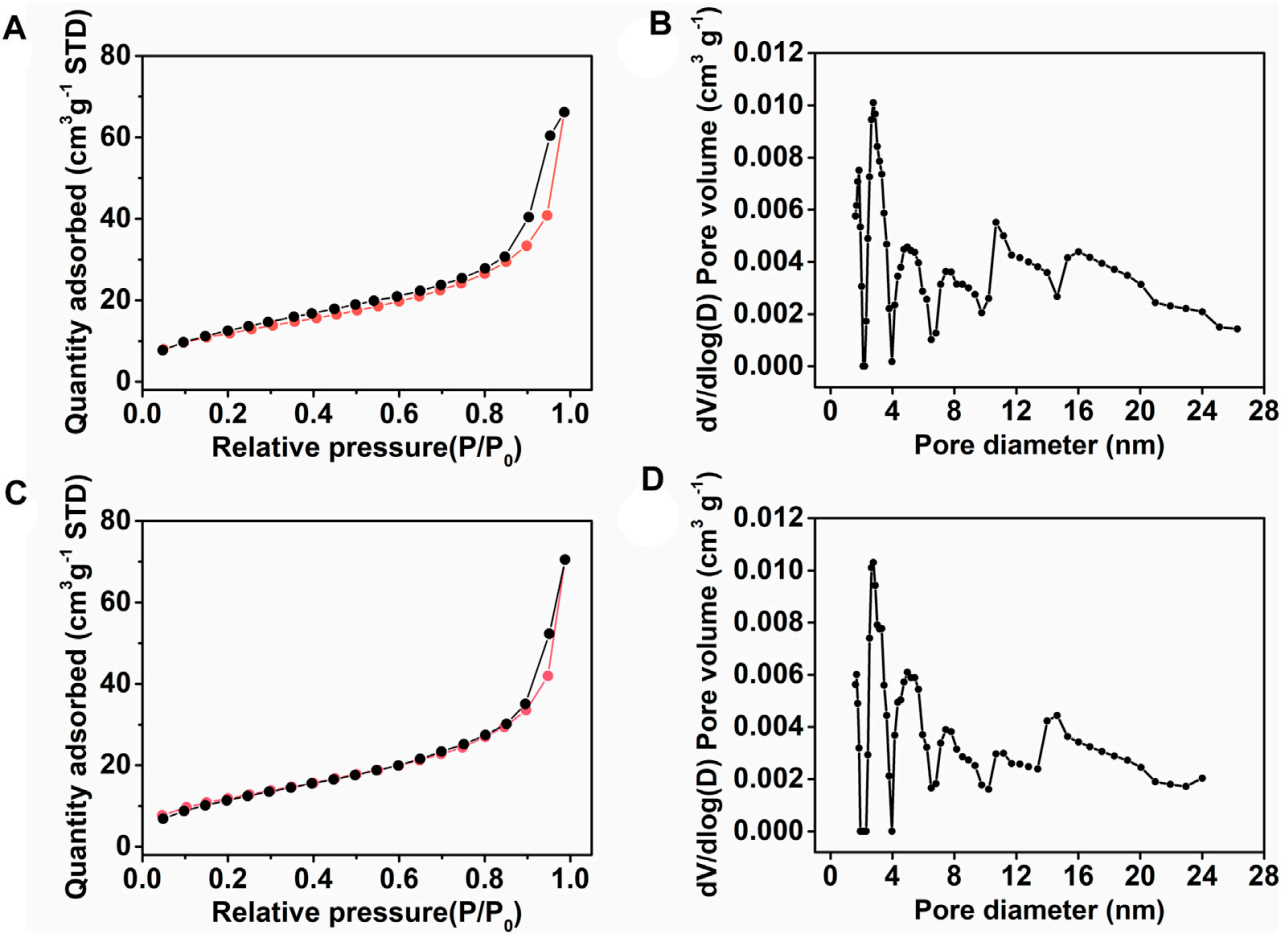
**(A,C)** Nitrogen adsorption/desorption isotherms and **(B,D)** corresponding pore size distribution plots of **(A,B)** monolith III and **(C,D)** monolith V, respectively.

On the other hand, nitrogen gas adsorption/desorption curves of the monoliths III and V were determined to evaluate their surface area and pore size distribution. From the results of nitrogen gas adsorption/desorption test, it could be attained that the surface areas of monolith III (Figure 2A) and monolith V (Figure 2C) were 43.2 m^2^ g^−1^ and 44.0 m^2^ g^−1^, respectively. Meanwhile, as observed from Figures 2B,D, the two monoliths exhibited a wider pore size distribution (3–25 nm), indicating a presence of mesopores with different sizes. Thus, the permeability, SEM, and nitrogen gas adsorption/desorption results confirmed that the hierarchical porous structure was successfully formed in monoliths III and V. The presence of macropores facilitate the flow of liquid, while the mesopores provide a large surface area, exposing more functional groups on the skeleton surface of the monolith.

**FIGURE 3:**
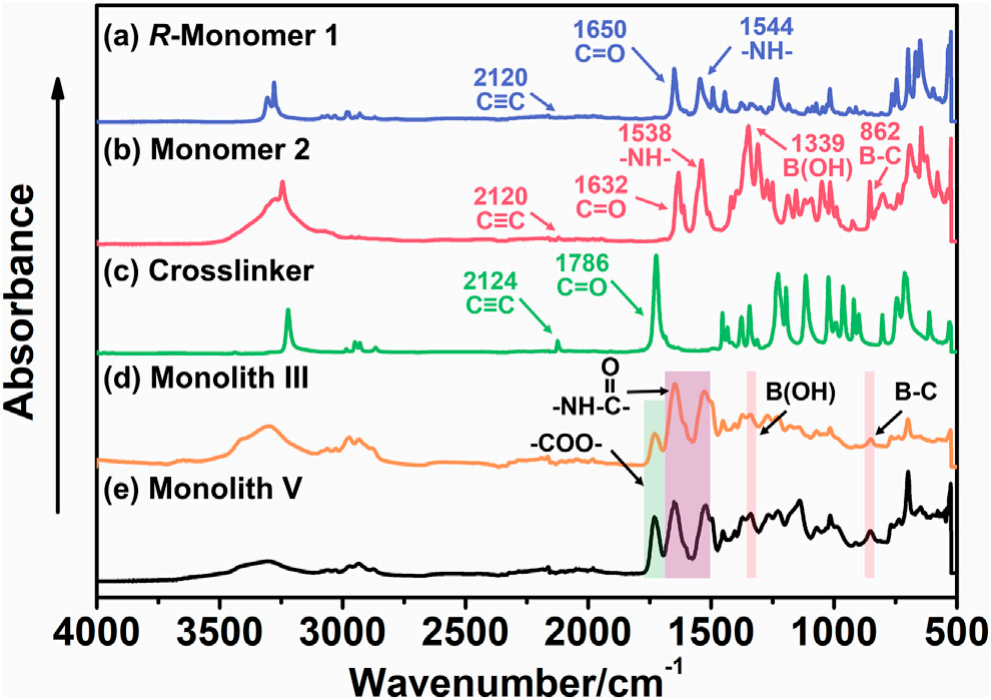
ATR/IR spectra of **(A)** chiral substituted acetylene (*R*-monomer 1, M1), **(B)** achiral substituted acetylene (monomer 2, M2), **(C)** crosslinker, **(D)** monolith III, and **(E)** monolith V.

To confirm the chemical structure and composition of the prepared monoliths and study the introduction of the boronic acid group, *R*-M1 (its spectrum was the same as *S*-M1), M2, the crosslinking agent, monolith III, and monolith V were characterized using ATR-IR (Figure 3). The peaks at ∼2,120 cm^−1^ in the spectra of *R*-M1 (Figure 3A), M2 (Figure 3B), and the crosslinker (Figure 3C) can be assigned to the stretching vibrations of the C≡C bond. It was observed that the intensity of these peaks decreased and even disappeared in the spectra of monoliths III (Figure 3D) and V (Figure 3E), indicating that the polymerization between the C≡C bonds was successful. Additionally, the strong peaks at ∼1,650 cm^−1^ and ∼1,544 cm^−1^ in the spectra of *R*-M1 and M2 were attributed to the stretching vibrations of C=O and N−H bonds, respectively, which confirmed the presence of the amide group. The signals at 1,339 and 862 cm^−1^ in M2 were ascribed to the characteristic peaks of the boronic group, and the strong peak at 1786 cm^−1^ in the spectra of the crosslinking agent was assigned to the stretching vibrations of the carbonyl group. These characteristic peaks were observed in the spectra of monoliths III and V, indicating that both the monomers and crosslinker sufficiently participated in the reaction. The ATR-IR results confirmed that the boronic group was successfully introduced into the materials, and the polymerization between the C≡C groups occurred during monolith formation.

**FIGURE 4:**
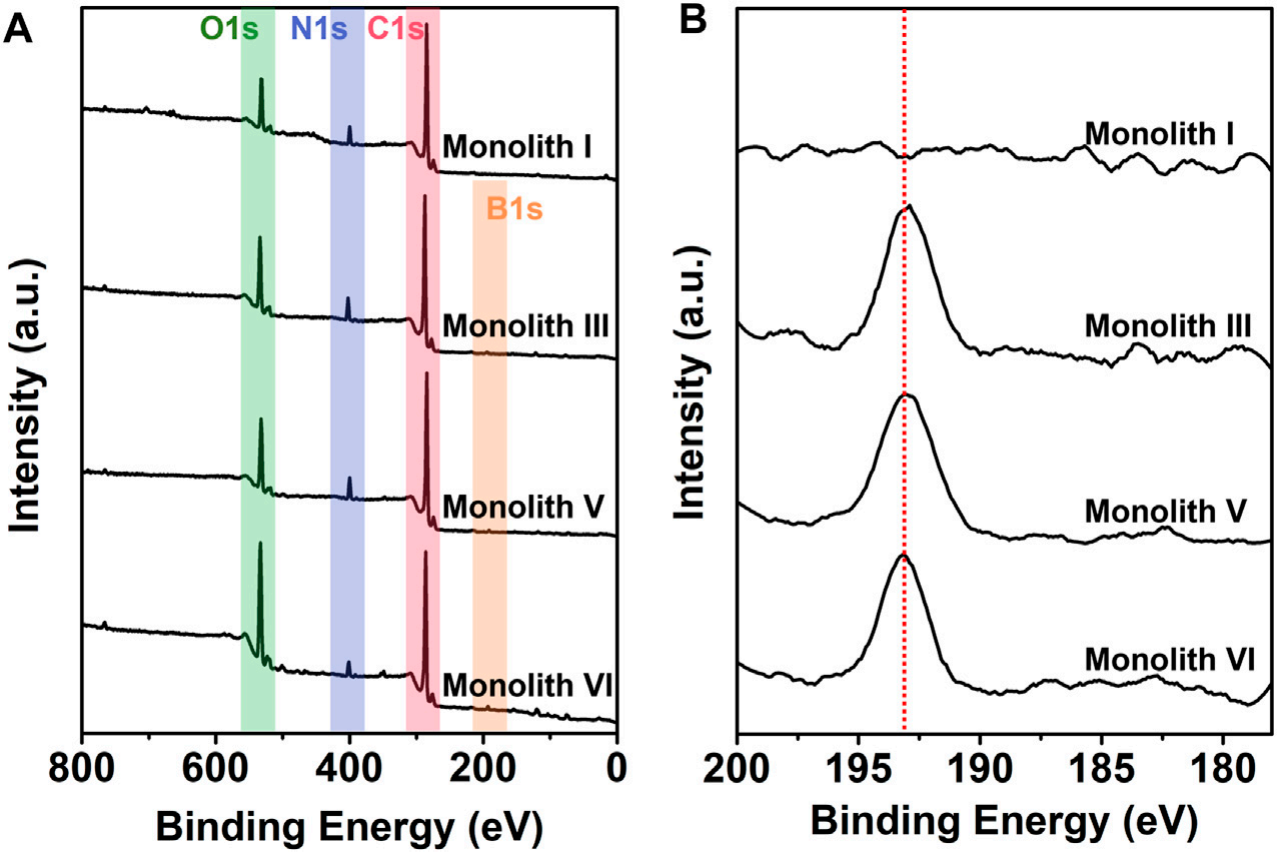
**(A)** Wide and **(B)** B 1s narrow X-ray photoelectron spectra of monoliths I, III, V, and VI.

Subsequently, monolith I (*R*-M1 and crosslinker), monolith III (*R*-M1, M2, and crosslinker), monolith V (*S*-M1, M2, and crosslinker), and monolith VI (M2 and crosslinker) were subjected to XPS to further characterize the chemical composition and investigate the successful introduction of boron on the surface of the monoliths after polymerization. As observed from the wide XPS spectra (Figure 4A), three elements (C, N, and O) were apparent on the surface of these four monoliths, while in the B 1s narrow spectra (Figure 4B), the characteristic signals of boron were observed in monoliths III, V, and VI, but not in monolith I (M2 was not added). The elemental atomic percentage (%) on the surface of the monoliths is shown in Table 2. At higher M2 content in the pre-polymerization solution, the atomic percentage of boron in the monoliths also increased. In addition, monoliths III and V showed almost equal boron content, suggesting that the preparation method can be universally applied to enantiomers with different conformations. The above results implied that the boronic group could be successfully introduced as a functional group on the surface of the porous structure in the monoliths *via* the preparation method to recognize the compounds containing *cis*-diol groups *via* reversible covalent bonds.

**TABLE 2:** The atomic percentage (%) on the surface of monoliths I, III, V and VI detected by X-ray photoelectron spectra.

	C	N	O	B
Monolith I	83.30	4.97	11.73	-
Monolith III	78.20	5.60	14.51	1.70
Monolith V	78.79	5.31	13.95	1.95
Monolith VI	71.27	3.61	22.10	3.02

**FIGURE 5:**
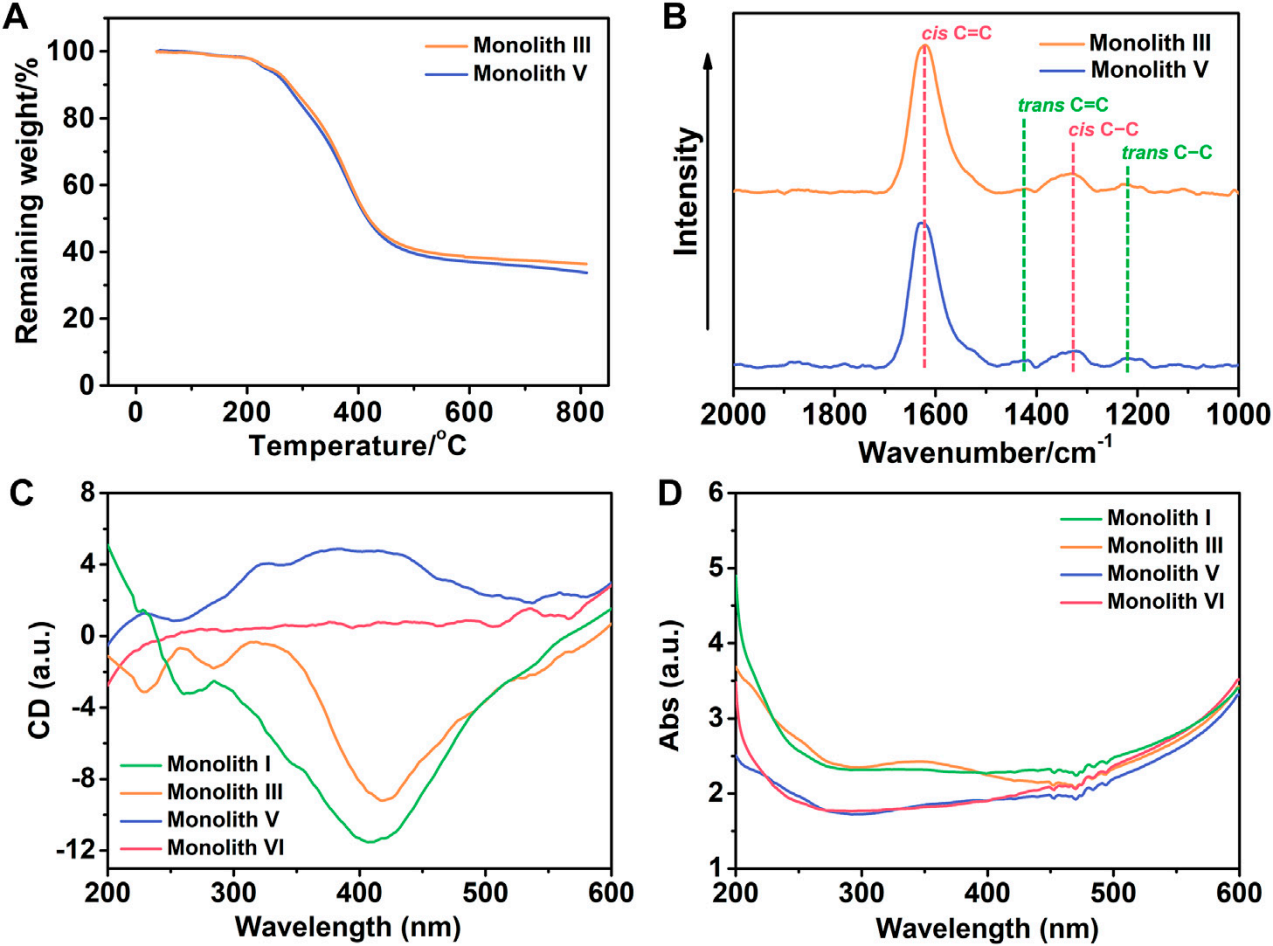
**(A)** Thermogravimetric analysis (TGA) curves of monoliths III and V. With a scanning rate of 10°C min^−1^, all the TGA curves were collected under a nitrogen atmosphere. **(B)** Raman spectra of monoliths III and V with the 532 nm excitation wavelength. **(C)** Circular dichroism (CD) and **(D)** ultraviolet–visible (UV−vis) absorption spectra of monoliths I, III, V and VI. The monoliths were respectively combined with KBr to compress into tablets to determine their spectra at 25°C.

TGA curves were collected to evaluate the thermal stability of the prepared monoliths III and V under nitrogen atmosphere. As observed from Figure 5A, there was significant weight loss from 216 to 600°C, and owing to the presence of boron, ∼35% residual weight remained (Figure 5A) in the two monoliths. This demonstrates that the monoliths possessed good thermal stability and the functional group was successfully introduced into the reaction system.

The main chain of the substituted polyacetylene is a conjugated polyene structure with alternating single and double bonds, in which the C=C bond in the main chain shows *cis* and *trans* configurations whose content significantly affects the tacticity of the main chain in the polymer. To ensure high tacticity of the polymer main chain, [(nbd)Rh^+^B^−^(C_6_H_5_)_4_] was selected as the catalyst in this case, and Raman spectra was employed to investigate the *cis* and *trans* content in monoliths III and V. As observed from Figure 5B, strong signals of *cis* C=C and C−C were observed at 1,623 and 1,336 cm^−1^, respectively, whereas the signals of *trans* C=C at 1,431 cm^−1^ and C−C at 1,213 cm^−1^ were very weak. This suggests that the main chain of the copolymer in monoliths III and V exhibited a higher *cis* content and tacticity, which facilitated the formation of the helical structure.

As previous literature reported, (Wang Y. et al., 2021) substituted polyacetylene synthesized by chiral substituted acetylene can form a helical conformation with single excess rotation due to conjugated structure of the main chain, which manifests optical activity. Conversely, achiral substituted acetylene without the chiral center easily forms substituted polyacetylene with a racemic helix conformation, with no optical activity. In this study, it was expected to fabricate a monolith that possess not only optical activity but also functional group, and thus chiral substituted polyacetylene (*R*- or *S*-M1) and achiral substituted polyacetylene (M2) with the phenylboronic acid group were selected. To verify whether the prepared monoliths exhibited optical activity, the CD and UV–vis absorption spectra were employed to investigate them. Because the monoliths cannot be dissolved using a common solvent, the mixture of the monoliths and KBr was compressed into tablets for qualitative measurements. As illustrated in Figure 5C, the negative signals near 400 nm appeared in the CD spectra of monolith I (prepared by *R*-M1 and crosslinker) and III (prepared by *R*-M1, M2 and crosslinker), while the positive signal at the same position appeared in monolith V (prepared by *S*-M1, M2 and crosslinker). However, there is no signal in the CD spectrum of monolith VI (prepared by M2 and crosslinker), indicating that the helical conformation with single excess rotation did not form owing to the absence of the chiral center. Moreover, the adsorption peaks also appeared at the corresponding positions in the UV–vis absorption spectra of monoliths I, III, and V as observed from Figure 5D. These results demonstrated that the different one-handed helical conformations respectively present in the monoliths III and V, which exhibited good optical activity even when achiral M2 participated in the polymerization with M1 to fabricate the copolymer.

### Investigation for Selectively Chiral Adsorption Ability of Monoliths

**FIGURE 6:**
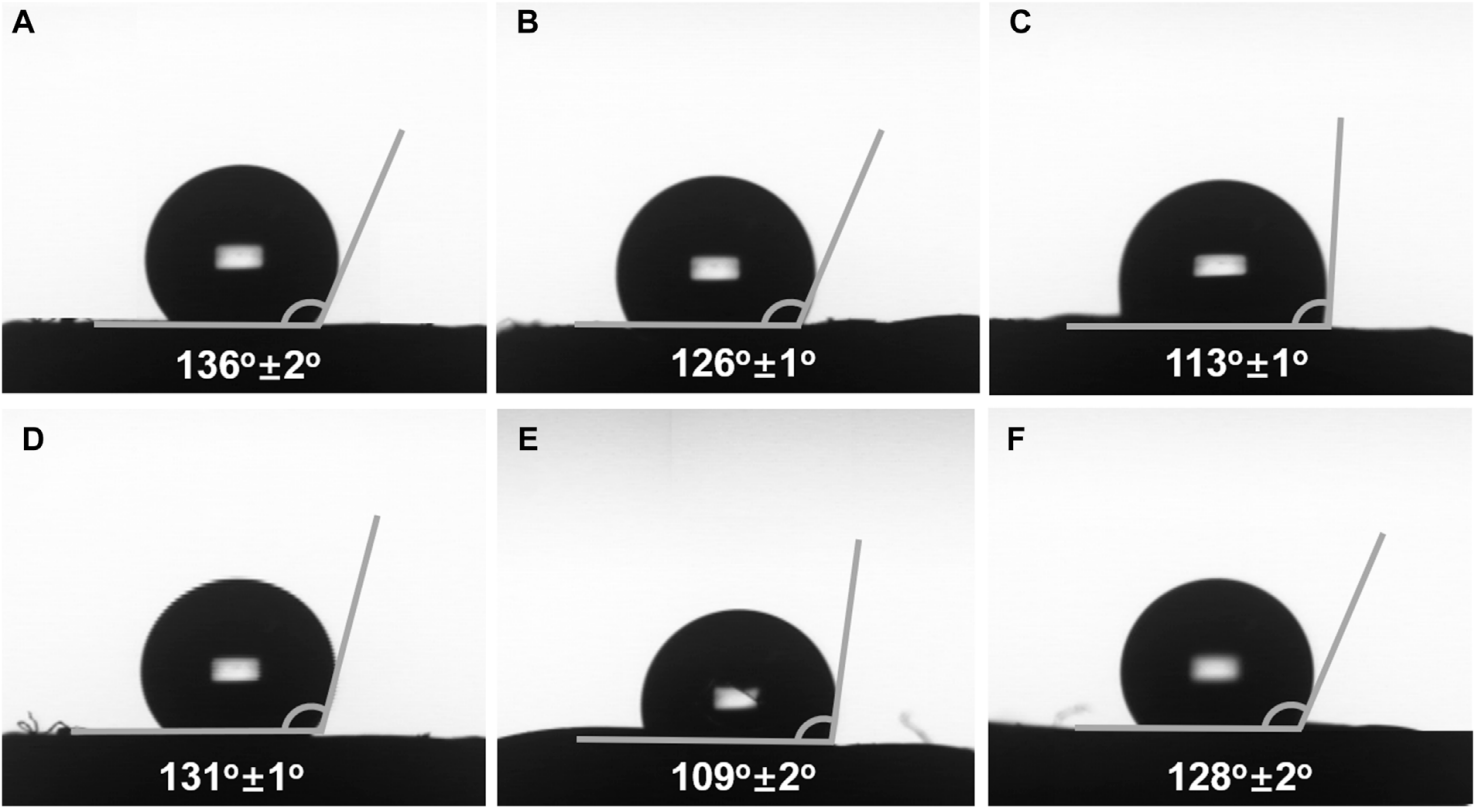
PB solution (pH = 8.6) contact angle on the surface of **(A)** monolith III and **(D)** monolith V. 10 mg mL^−1^ α-*D*-glucose PB solution contact angle on the surface of **(B)** monolith III and **(E)** monolith V. 10 mg mL^−1^ α-*L*-glucose PB solution contact angle on the surface of **(C)** monolith III and **(F)** monolith V.

As above mentioned, the resulting monoliths simultaneously possessed chirality and boronic acid group, indicating that they may show selectivity for the target compound with chirality and *cis*-diol group. α-Glucose has *cis*-diol group, and thus it could be adsorbed by the materials with boronic acid group to form reversible covalent bonds. Moreover, α-glucose has two conformations including α-*D*- and α-*L*-glucose, which can interact with chiral materials, such as helical polymer. Therefore, the pair of enantiomers was chosen to investigate the selectively chiral adsorption ability of the resulting monoliths. The contact angle test was used to determine contact angle of three different solutions (PB solution (pH = 8.6), 10 mg ml^−1^ α-*D*-glucose PB solution, and 10 mg ml^−1^ α-*L*-glucose PB solution) on monoliths III and V. As observed from Figures 6A,D, the PB solution contact angles on monoliths III (prepared by *R*-M1, M2 and crosslinker) and V (prepared by *S*-M1, M2 and crosslinker) were respectively 136°±2° and 131°±2°. However, when the 10 mg ml^−1^ α-*D*- and α-*L*-glucose PB solutions were used as samples, the values of contact angles on the two monoliths were decreased owing to the existence of boronic acid group. Even more to the point, α-*D*-glucose PB solution contact angle on monolith III was 126°±1° in Figure 6B, which was higher than that of α-*L*-glucose PB solution (113°±1°) in Figure 6C. On the contrary, α-*D*-glucose PB solution contact angle on monolith V was 109°±2° (Figure 6E), and α-*L*-glucose PB solution contact angle increased to 128°±2° in (Figure 6F). The phenomena implied that monolith III has stronger interaction with α-*L*-glucose compared to α-*D*-glucose owing to matching chiral conformation, and monolith V showed a opposite performance. It could be demonstrated that the synthesized monoliths have recognition ability for α-glucose with different conformation.

Meanwhile, 0.5 mg ml^−1^ α-*D*- and α-*L*-glucose PB solution (pH = 8.6) were chosen to investigate the selectively chiral adsorption ability of the resulting monoliths. The water contact angle test was used to determine the change in water contact angle of monoliths before and after adsorbing α-*D*- and α-*L*-glucose. In Supplementary Figure S1 (Supporting information), it can be found that the water contact angles of monoliths III and V were greater than 120°, indicating that they are hydrophobic. After adsorbing α-*D*- and α-*L*-glucose, the water contact angles of monolith III respectively decreased to 75°±3° (Supplementary Figure S1B) and 43°±2° (Supplementary Figure S1C), and monolith V respectively changed to 39°±2° (Supplementary Figure S1E) and 47°±3° (Supplementary Figure S1F). It indicated that more amount of α-*L*-glucose was adsorbed on the surface of monolith III compared to α-*D*-glucose, and more amount of α-*D*-glucose was adsorbed on the surface of monolith V in the same condition. These results could prove that the synthesized monoliths have selectively chiral adsorption ability for the target compound with chirality and *cis*-diol group and the potential in chiral separation or adsorption.

## Conclusion

Using the CPIPS method, hierarchically porous boronic acid group-functionalized monoliths with optical activity were successfully synthesized. Chiral substituted acetylene and achiral substituted acetylene with the boronic acid functional group were used as monomers in the presence of a catalyst ([(nbd)Rh^+^B^−^(C_6_H_5_)_4_]), crosslinker, and porogenic solvent (THF and CH_3_OH). The macropores size could be regulated by changing the composition of the pre-polymerization solution. Moreover, analysis by SEM and nitrogen gas adsorption/desorption isotherms indicated that the monoliths possessed a large surface area and hierarchically porous structure. Further, ATR-IR and XPS spectra confirmed that the boronic acid functional group was successful introduced on the surface of the porous structure in the resulting monolith. Furthermore, TGA confirmed that the monoliths were thermally stable. Most importantly, the polymerization occurred in the substituted acetylene and was initiated by the rhodium catalyst; thus, the main chain of the copolymer that constituted the monoliths showed a high *cis* content, as per Raman spectra, indicating a high tacticity. In the case where both the enantiomers (*R*-M1 and *S*-M2) were used as monomers, reverse signals in the CD spectra derived from the formation of the chiral helical substituted polyacetylene were observed, revealing that the monoliths had good optical activity. The optical activity and boronic acid functional group in the monolith can interact with target compound with chirality and *cis*-diol group. These results clearly indicate that the monoliths have the potential for various applications, such as chiral recognition, chiral controlled release, and selectively chiral adsorption.

## Data Availability

The original contributions presented in the study are included in the article/Supplementary Material, further inquiries can be directed to the corresponding author.
