# Supplementary material for: Facile Fabrication of Hierarchically Porous Boronic Acid Group-Functionalized Monoliths With Optical Activity for Recognizing Glucose With Different Conformation
Source: Front Chem. 2022 Jun 8;10:939368. doi: 10.3389/fchem.2022.939368 (PMC9213758; doi:10.3389/fchem.2022.939368)
Supplement: Supplementary file 1 [file DataSheet1.docx]

Supplementary Material

Facile fabrication of hierarchically porous boronic acid group-functionalized monoliths with optical activity for recognizing glucose with different conformation

Yan Wang, Luwei Zhang, Yu-I Hsu*, Taka-Aki Asoh, Hiroshi Uyama*

Department of Applied Chemistry, Graduate School of Engineering, Osaka University, 2-1 Yamadaoka, Suita, Osaka, 565-0871, Japan

^*^ To whom correspondence should be addressed:

Prof. Yu-I Hsu

Tel: +81-6-6879-7365

Fax: +81-6-6879-7367

E-mail: yuihsu@chem.eng.osaka-u.ac.jp

Prof. Hiroshi Uyama

Tel: +81-6-6879-7364

Fax: +81-6-6879-7367

E-mail: uyama@chem.eng.osaka-u.ac.jp


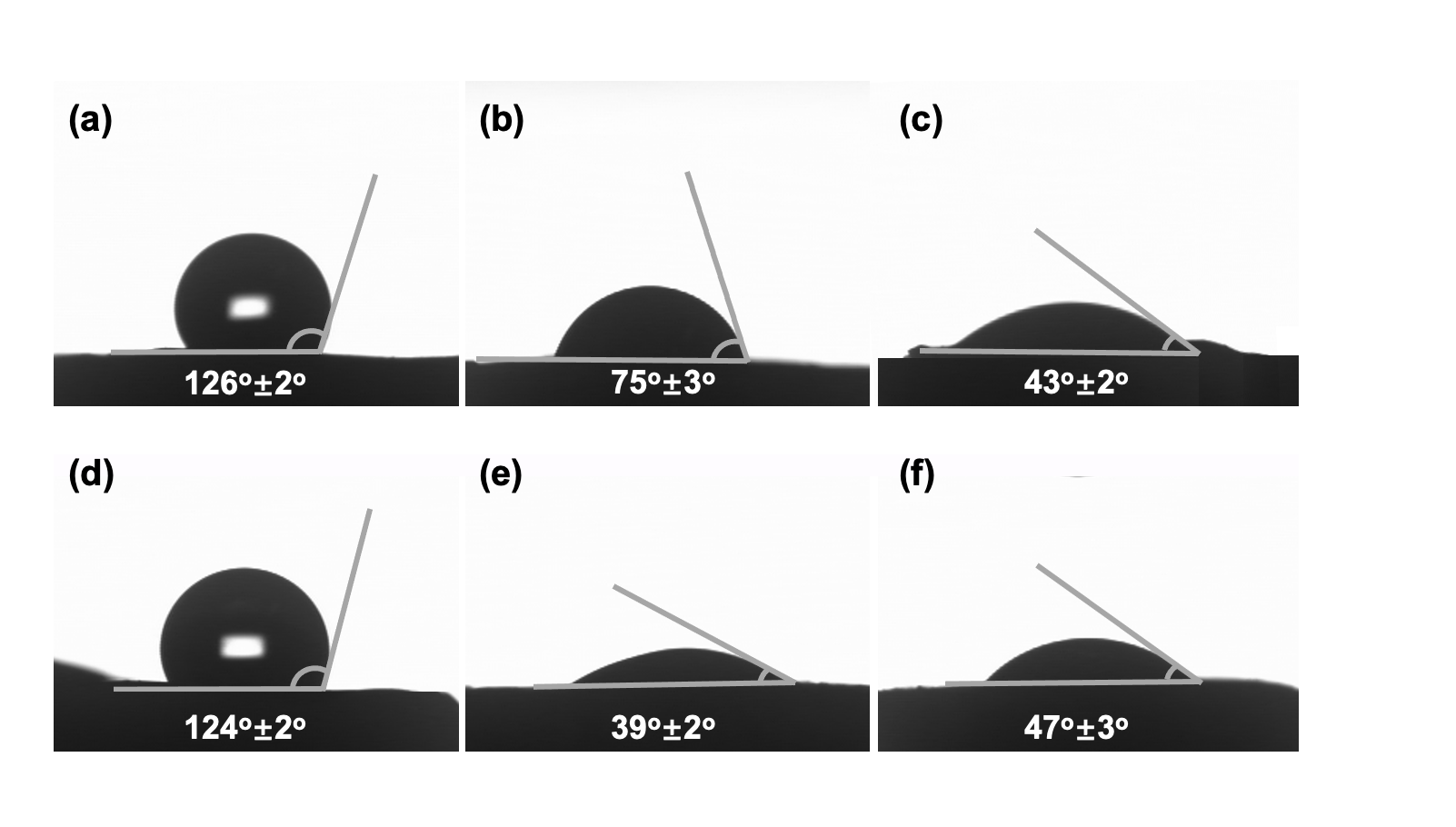


**Figure S1** Water contact angle of the surface of (a) pristine, (b) adsorbed α-*D*-glucose, and (c) adsorbed α-*L*-glucose monolith III, and (d) pristine, (e) adsorbed α-*D*-glucose, and (f) adsorbed α-*L*-glucose monolith V.
